# Shifting Perceptions: Breaking the Stigma

**DOI:** 10.1192/bjo.2026.11280

**Published:** 2026-06-30

**Authors:** Aishath Shahama, Jerome Carson

**Affiliations:** University of Greater Manchester, Bolton, Greater Manchester, United Kingdom

## Abstract

**Aims:**

A community engagement programme can improve individuals’ mental health literacy and create awareness about mental health in communities. The conference aimed to reduce mental health stigma and improve mental health outcomes in the community.

**Methods:**

The impact of the conference was mapped out by applying Theory of Change. They are input, which are resources including financial sponsorship and institutional support; expertise from leading psychiatrists in the UK, psychologists, researchers, practitioners, and individuals with lived experiences from a South Asian background. Keynotes were given by Professors Dinesh Bhugra, Nusrat Husain, and Subodh Dave. Engagement tools include a survey (N=42), sticky note messages (N=41), a data map, and a website (https://samhstigma.my.canva.site). Activities included 15 presentations, both in person and online. Outputs included hybrid delivery and participation (62 in-person, 13 online), recording of the conference, and a booklet with presenters’ profiles and a summary of their presentation.

**Results:**

Short-term outcomes include increased mental health literacy, awareness of the cultural dimension of stigma, attitudinal shift, and strengthened professional networking. Some medium-term outcomes included greater collaboration between institutions and the community, integration of lived experience voices into future research and practice, through ripple effects of awareness, increased help-seeking behaviour, and encouraging culturally sensitive approaches being adopted in clinical settings. Long-term outcomes included, at a personal, community, and institutional level, reducing stigma, access to mental health services improved for South Asians.

**Conclusion:**

“*Nothing About Us Without Us*” can further strengthen the Theory of Change framework applied to the community engagement program. It aligns with the critical long-term impact, which is empowerment and inclusion. It makes community engagement more effective and relatable as it enhances credibility and authenticity. The principle suggests that people who are directly affected by an issue must be actively involved in making decisions, policies, and programmes about that particular issue. Bilateral learning is another principle that is integrated into the programme. It involves creating a two-way exchange of knowledge between the community and professionals, including psychiatrists, counselling therapists, advocates, and researchers. The community gains information about evidence-based strategies for mental health care.

In the future, community members can be included in co-designing the agenda for community engagement programmes. The conference format can further be made interactive by including a panel discussion where queries from the community are clarified by mentalhealth professionals, conducting a workshop on shared problem-solving, storytelling, and allocating time for questions and answers with people with lived experience, alongside reflections from the professionals.

